# The Role of the Extracellular Matrix in Neural Progenitor Cell Proliferation and Cortical Folding During Human Neocortex Development

**DOI:** 10.3389/fncel.2021.804649

**Published:** 2022-01-24

**Authors:** Katherine R. Long, Wieland B. Huttner

**Affiliations:** ^1^Centre for Developmental Neurobiology, Institute of Psychiatry, Psychology and Neuroscience, King’s College London, London, United Kingdom; ^2^MRC Centre for Neurodevelopmental Disorders, King’s College London, London, United Kingdom; ^3^Max Planck Institute of Molecular Cell Biology and Genetics, Dresden, Germany

**Keywords:** extracelluar matrix, human, neocortex, development, neural progenitor

## Abstract

Extracellular matrix (ECM) has long been known to regulate many aspects of neural development in many different species. However, the role of the ECM in the development of the human neocortex is not yet fully understood. In this review we discuss the role of the ECM in human neocortex development and the different model systems that can be used to investigate this. In particular, we will focus on how the ECM regulates human neural stem and progenitor cell proliferation and differentiation, how the ECM regulates the architecture of the developing human neocortex and the effect of mutations in ECM and ECM-associated genes in neurodevelopmental disorders.

## Introduction

The human brain has long been known to contain a large amount of extracellular matrix (ECM) and ECM-associated molecules (Sanes, 1983, 1989). In the adult, this equates to roughly 20% of the total brain volume, but during fetal development, it is double that amount, at roughly 40% (Jovanov Milošević et al., 2014). Given its abundance, it is a little surprising that we do not fully understand the role of the ECM in the development of the neocortex. In fact, we do not yet know exactly which ECM components are expressed throughout development. We have learnt a lot about the role of ECM in neural development from many different animal and *in vitro* model systems (expertly reviewed elsewhere; Bandtlow and Zimmermann, 2000; Zimmermann and Dours-Zimmermann, 2008; Marthiens et al., 2010; Barros et al., 2011; Franco and Müller, 2011; Long and Huttner, 2019; Amin and Borrell, 2020). This includes roles in neural progenitor proliferation, differentiation, morphology, axonal and dendritic elongation and connectivity, neuronal migration and cortical folding. It has also been suggested that the ECM can create functional microdomains within the developing neocortex, for example by regulating the diffusion of signalling factors, restricting them to a discrete area, or by altering the migration route of newborn neurons (Dityatev et al., 2010). However, much of this data has been generated in animal models and it remains unclear how much can be extrapolated to human neocortex development. Major differences have been reported for human and non-human primate neocortex development in comparison to mouse development. For example, RNA-sequencing studies have revealed important differences in ECM expression between the developing human neocortex compared to the embryonic mouse neocortex (Fietz et al., 2012; Miller et al., 2014; Florio et al., 2015). One of the most striking of these differences is that significantly more ECM is expressed in the human fetal neocortex (Fietz et al., 2012). This raises the question as to whether this ECM has played an important role in the evolution of the human neocortex.

In this review, we will discuss the role of the ECM in human and non-human primate neocortex development, focusing on neural progenitors, neocortical tissue architecture and neurodevelopmental disorders.

## ECM and Neural Progenitors

ECM and ECM receptors have long been known to regulate the behaviour of neural progenitor cells (NPCs; Loulier et al., 2009; Radakovits et al., 2009; Fietz et al., 2010; Barros et al., 2011; Long et al., 2016; Long and Huttner, 2019; Amin and Borrell, 2020). However, compared to the large volume of evidence of ECM function in animal models, the data in human and non-human primate neocortex is limited.

### ECM Expression and Function

**Figure 1:**
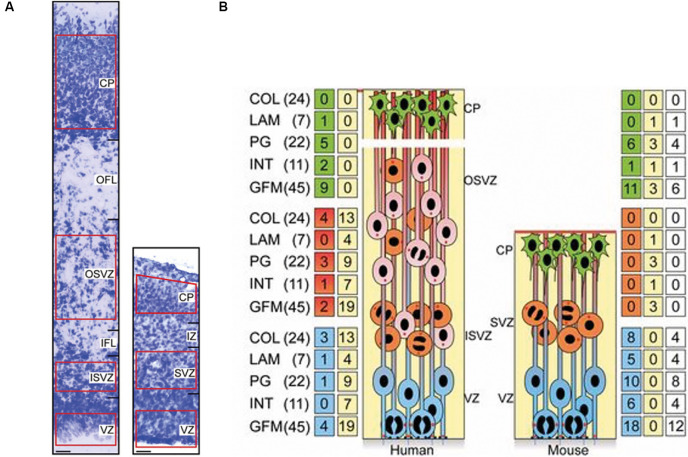
Increased expression of extracellular matrix (ECM) components in the proliferative zones of the human fetal neocortex compared to the embryonic mouse neocortex. **(A)** Nissl staining of the cortical wall of a 15 post-conception week (pcw) human fetal neocortex (left) and of an embryonic day (E) 14.5 embryonic mouse neocortex (right). Scale bars represent 20 μm. The areas used for transcriptomic analysis are highlighted in red. The different zones of the cortical wall are labelled as ventricular zone (VZ), subventricular zone (SVZ), inner SVZ (ISVZ), outer SVZ (OSVZ), inner fibre layer (IFL), outer fibre layer (OFL), intermediate zone (IZ) and cortical plate (CP). **(B)** Schematic illustrating the major cell types within the developing cortical wall (APs = blue, BPs = orange and red, neurons = green) and the distribution of ECM and ECM-associated components within these areas. The major families of ECM components are the collagens (COL), laminins (LAM), proteoglycans (PG), integrins (INT) and growth factors/morphogens (GFM). Numbers in parentheses indicate the total numbers of genes within each family. Numbers in the green/red/blue boxes indicate the number of genes overexpressed in the adjacent area. Numbers in the yellow boxes indicate the number of genes that are overexpressed in more than one human or mouse zone. Numbers in the white boxes indicate the number of genes that are overexpressed in both humans and mice. The cells within the zones are: APs are located within the VZ in both mice and humans, BPs are located in the SVZ in mice, and within the ISVZ and OSVZ in humans, neurons are located in the CP in both species. Adapted from Fietz et al. (2012).

The RNA sequencing studies described above have suggested that many ECM components and receptors are expressed in the developing human neocortex (Figure 1; Fietz et al., 2012; Miller et al., 2014; Florio et al., 2015). This includes the major family of ECM receptors, the integrins (Hynes, 2002). Downstream of the integrins lie many important signalling pathways already known to regulate aspects of neural development, including PI3-kinase and Akt for cell survival, ERK and CyclinD1 for proliferation, and cdc42 and rac for cell migration (Hynes, 2002). The role of these factors, and others such as integrin-linked kinase and focal adhesion kinase, have been well studied in animal models (Beggs et al., 2003; Niewmierzycka et al., 2005; Tsuda et al., 2010; Valiente et al., 2011; Long et al., 2016), but less is known about their function in the developing human neocortex. However, more is known about the integrins themselves. In particular, integrins alpha 6 and beta 1 are known to be markers of human neural stem cells (NSCs; Hall et al., 2006). This integrin combination is able to bind to two of the main families of ECM components, the laminins and the collagens.

The most well-known of the laminins is laminin-111, which is highly expressed in both early mouse and human neocortex but is down-regulated later in development (Barros et al., 2020). The roles of laminins in mouse neocortex development are highly varied (Haubst et al., 2006; Loulier et al., 2009; Radakovits et al., 2009; Güven et al., 2020), but their roles in human neocortex development are yet to be fully investigated. Current evidence has shown that plating human NSCs onto laminin *in vitro* promotes both neurogenesis of the NSCs and then neurite outgrowth from the newborn neurons (Flanagan et al., 2006; Ma et al., 2008). However, in contrast, laminin has also been shown to promote the proliferation of human NSCs (Flanagan et al., 2006; Hall et al., 2008). Indeed, laminin-derived peptides, such as RGD, can promote both NSC proliferation and differentiation *in vitro* (Li et al., 2014), suggesting the function of laminins is highly complex and may be context-dependent, as seen in the mouse neocortex (Haubst et al., 2006; Loulier et al., 2009; Radakovits et al., 2009; Güven et al., 2020).

Other ECM components have also been reported to promote proliferation of human NSCs/NPCs, such as collagen IV (Hubert et al., 2009) and heparan sulphate proteoglycans (HSPGs; Oikari et al., 2016; Yu et al., 2017). However, as with laminins, these ECM components have also been implicated in neural differentiation (Oikari et al., 2016; Okolicsanyi et al., 2018). HSPGs are part of the proteoglycan family, which also includes the chondroitin sulphate proteoglycans, syndecans and glypicans. They have been shown to regulate several aspects of neural development, particularly neuronal migration (Bandtlow and Zimmermann, 2000; Ishii and Maeda, 2008; Maeda, 2015). The majority of these findings have been reported in 2D *in vitro* experiments using human NSCs/NPCs, but very few studies have investigated their function within an intact tissue environment. As ECM components often interact with each other and the surrounding tissue, their functions may differ between these simplified 2D environments and the complex 3D environment of the neocortex.

To address this, several studies have focused on understanding the specific network of ECM components that are important for human neocortex development. Fietz and colleagues showed that multiple ECM components were more highly expressed in the developing human neocortex compared to mouse (Figure 1; Fietz et al., 2012). This was particularly so in regions of the cortical wall that contained highly proliferative progenitors. In the mouse neocortex, this was the ventricular zone, which contains proliferative apical progenitors (APs). Within these APs, ECM was more highly expressed in proliferative progenitors in comparison to progenitors about to undergo differentiative divisions (Arai et al., 2011). However, in the human neocortex, ECM expression was not only high in the ventricular zone but was also high in both the inner and outer subventricular zones, which contain proliferative basal progenitors (BPs; Fietz et al., 2012). This suggested a pro-proliferative role of the ECM in the developing neocortex. Further analysis indicated the regulation of this ECM could be controlled by the transcription factor SOX9. SOX9 was found to only be expressed in mouse APs but was expressed in both APs and BPs in the human fetal neocortex (Güven et al., 2020). Additionally, SOX9 over-expression in the embryonic mouse neocortex resulted in an increase in BP proliferation and increased ECM expression (Güven et al., 2020).

This proliferative role of the ECM is further supported by the expression and activation of integrins in BPs. Integrin alpha v beta 3 is more highly expressed in the proliferative human BPs compared to the less proliferative mouse BPs (Stenzel et al., 2014), consistent with the above reports of higher ECM expression in the former cells (Fietz et al., 2012; Miller et al., 2014; Florio et al., 2015). When integrin alpha v beta 3 was activated in the embryonic mouse neocortex, by addition of a function-activating antibody, this increased BP proliferation (Stenzel et al., 2014). Integrin beta 1 activation is also important for human BP proliferation, as blocking integrin beta 1 function in human fetal neocortex slice cultures decreased BP proliferation (Kalebic et al., 2019). The expression and function of integrins in human BPs is thought to be important for the interaction of these cells with their pro-proliferative ECM environment, enabling their increased proliferative capacity compared to BPs in the mouse. Human BPs have a more complex morphology in comparison to mouse, with more variation and a higher number of radial processes. These processes contain integrins that are able to interact with the surrounding ECM. Therefore, the more radial processes, the more pro-proliferative signals the BP can receive from the ECM environment (Kalebic et al., 2019; Kalebic and Huttner, 2020).

### Models to Study ECM

A major reason for our limited knowledge of the role of the ECM in human and non-human primate neocortex development is the difficulty in modelling primate neocortex development. There are several options currently available, including the use of primary tissue or the generation of 2D and 3D models, notably brain organoids, from embryonic and induced pluripotent stem cells (ESCs and iPSCS). Primary tissue studies have provided valuable insight, as described above (Kalebic et al., 2019) and in the following section in more detail (Long et al., 2018). This includes the use of primary tissue to study ECM expression patterns (Fietz et al., 2012; Jovanov Milošević et al., 2014; Miller et al., 2014; Florio et al., 2015; Long et al., 2018) and the use of *ex vivo* tissue culture techniques to study the role of the ECM in an intact human tissue environment (Long et al., 2018; Kalebic et al., 2019; Eigel et al., 2021). However, access to this tissue is limited, making the 2D and 3D models from ESCs and iPSCs an attractive alternative for many researchers.

**Figure 2:**
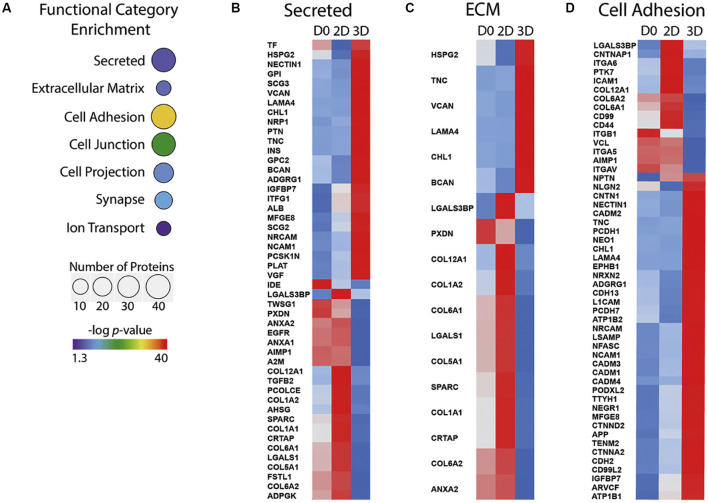
ECM components expressed differ between 2D and 3D *in vitro* cultures of human iPSCs undergoing differentiation to NPCs. **(A)** Functional categories that are significantly over-represented in 3D iPSC to NPC differentiation, selected from proteomic analysis of 2D and 3D cultures. The number of proteins is indicated by sphere size, the *p*-value is indicated by colour. **(B–D)** Heatmaps showing protein abundance profile at day 0 (D0) of culture, day 30 of2D culture (2D) and day 30 of 3D culture (3D). *Z* scores are colour-coded from blue (downregulation) to red (upregulation). Secreted proteins **(B)**, ECM **(C)** and cell adhesion protein **(D)** categories are shown. Adapted from Simão et al. (2018). iPSC, induced pluripotent stem cell; NPC, neural progenitor cell.

Using these 2D and 3D models comes with many clear advantages. They allow the study of human and non-human primate cells, and recent protocols have shown early neocortex development can be recapitulated relatively well in these *in vitro* systems (Mason and Price, 2016; Jabaudon and Lancaster, 2018; Heide et al., 2018; Kanton et al., 2019; Kyrousi and Cappello, 2019; Molnár et al., 2019). However, how these cells are cultured may have a significant effect on the investigation of ECM function in these models. For example, the ECM generated *in vitro* by human iPSC-derived NPCs is different in 2D cultures compared to 3D culture systems (Figure 2). The ECM is more similar to the *in vivo* composition when iPSC-derived NPCs are culture in 3D neurospheres than 2D systems, containing more proteoglycans and fewer basement membrane components (Figure 2; Simão et al., 2018). When these cells are grown as more complex cerebral organoids, the ECM composition expressed by the APs is indeed more similar to those of APs in the human fetal neocortex (Camp et al., 2015). This includes the ECM components collagen IV alpha 5, laminin alpha 1 and brevican, and the ECM receptors integrin alpha 6 and beta 8.

Additionally, the non-neuronal cells within the organoids also express ECM found in the fetal neocortex, including decorin, lumican, and collagen I alpha 2 (Camp et al., 2015). This data would suggest that 3D *in vitro* models, such as cerebral organoids, are better suited for the study of the role of ECM in neocortex development. However, although these 3D culture systems promote expression of some ECM components, there are still many that are not expressed that may play important roles. These include those secreted from cell types not present in these organoids, such as the blood vessels and meninges. It would be interesting to investigate how the addition of the ECM generated by these cells affects the development of such cerebral organoids.

One way to study the entire ECM network in 3D cell-based models is to use ECM extracted from primary tissue. Human iPSCs grown on ECM secreted by NPCs increased the expression of neuronal genes, such as tubulin beta 3, in comparison to ECM secreted by ESCs (Yan et al., 2015). ECM extracted from adult human brain tissue promoted increased laminin deposition in cerebral organoids, increased proliferation of APs and increased neural production (Cho et al., 2021). It would be interesting to investigate the effect of ECM extracted from fetal neocortex tissue using this model, as it is highly likely to differ in composition to the adult ECM extract used in this study.

How the ECM composition can direct the neurogenesis of iPSC-derived NPCs is yet to be determined. In addition to the known signalling functions of ECM, such as activation of integrins, the stiffness and elasticity of the matrix can also alter neurogenesis. Human iPSCs grown in softer ECM matrices differentiate into neuroectoderm and generate neurons, unlike iPSCs grown on stiffer matrices (Keung et al., 2012). This raises an interesting issue when comparing 2D and 3D *in vitro* models, as 3D models will inherently generate environments that are softer than 2D cultures grown on glass or plastic. How stiffness changes in these culture systems and how this compares to tissue will be important to determine the different roles of distinct ECM compositions in human neocortex development.

## ECM and Neocortex Tissue Architecture

The expression and function of ECM have also been studied in human fetal neocortex tissue. ECM expression appears to be dynamic, with changes in the cortical wall over the course of development. For example, hyaluronic acid expression levels change with the laminar structure of the subplate between 13 and 21 post-conception weeks (Kostović et al., 2019). Temporal and spatial changes in ECM expression could have a major impact on the development of the neocortex and has been hypothesised to affect the folding of the cortical plate (van Essen, 2020). This could occur in several ways, including mechanical forces and neuronal migration.

**Figure 3:**
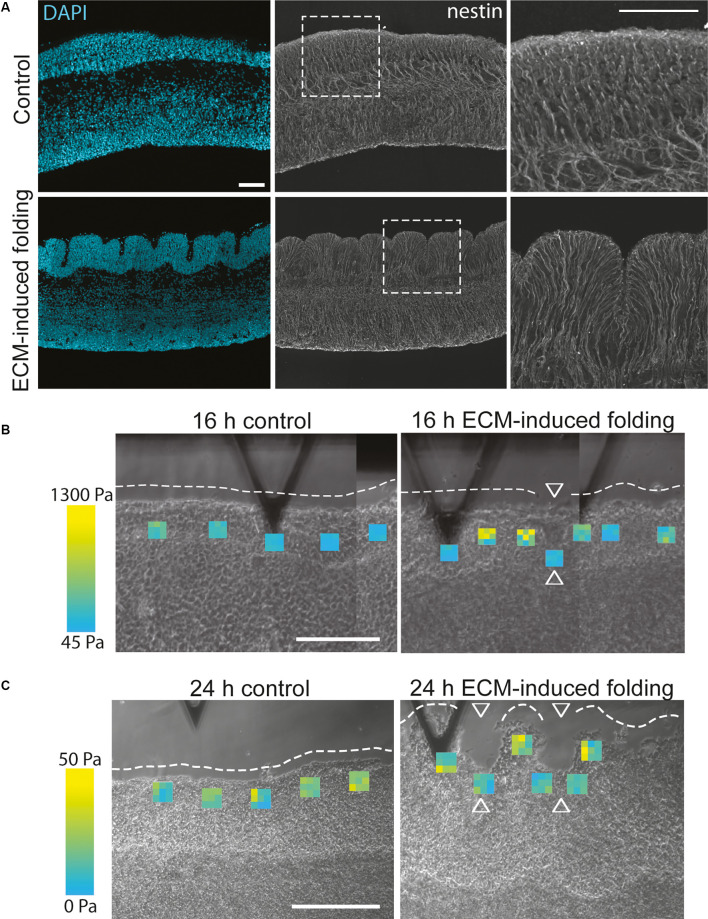
ECM can induce folding and changes in ECM stiffness in human fetal neocortex *ex vivo* cultures. **(A)** Images showing a 13 gestation week (GW) human fetal neocortex after 24 h of *ex vivo* culture in the control condition (upper panels) or after the addition of the ECM components HAPLN1, lumican and collagen I (lower panels), with DAPI staining (blue) and immunofluorescence for the radial glial process marker nestin (grey). The addition of these ECM components induced the folding of the cortical plate. White dashed boxes delineate areas shown in the panels on the right. Scale bar represents 50 μm. **(B,C)** Images showing atomic force microscopy of 13 GW human fetal neocortex after 16 h (**B**, folding ongoing) or 24 h (**C**, folding complete) of *ex vivo* culture in control conditions (left panels) or after the addition of the ECM components HAPLN1, lumican and collagen I (right panels, ECM-induced folding). Each heatmap square shows nine Young’s modulus values in the position the measurement was taken (see the range of values from blue (lowest) to yellow (highest)). White dashed lines outline the unfolded or folded CP surface; arrowheads indicate sulci. The stiffest measurements (yellow) are found in the forming gyri compared to the softer measurements (blue) in the forming sulci. Scale bar represents 200 μm. Adapted from Long et al. (2018).

ECM composition has already been shown to affect the stiffness of human fetal neocortex slice cultures, as removing specific ECM components (hyaluronic acid) using enzymatic degradation (by hyaluronidases) resulted in reduced stiffness (Long et al., 2018). In addition, cortical folding could be induced in these human fetal neocortex slice cultures by adding the ECM components Hyaluronan And Proteoglycan Link Protein 1 (HAPLN1), lumican, and collagen I (Figure 3; Long et al., 2018). This ECM-induced folding was also replicated in sliced neocortical organoids (Qian et al., 2020). In the primary tissue, the ECM-induced folding required an increase in ECM stiffness prior to the fold formation, resulting in a specific pattern of stiffer ECM at the gyrus of the fold and softer ECM in the sulcus (Figure 3; Long et al., 2018). Although tissue and ECM mechanics are hypothesised to regulate cortical folding (Karlinski and Reiner, 2018), this ECM-induced folding also required ERK signalling downstream of the hyaluronic acid receptor CD168 (Long et al., 2018), suggesting the role of ECM in cortical folding and tissue morphology will require both mechanical changes and signalling *via* ECM receptors.

ECM composition was also hypothesised to affect the migration of neurons. In the mouse, the majority of glutamatergic neuronal migration in the cortex is thought to occur radially, with neurons migrating along the radial glia scaffold up to the cortical plate. However, tangential migration of glutamatergic neurons has been observed in the developing macaque cortex (Cortay et al., 2020) and is thought to be important for cortical folding (van Essen, 2020). It has been hypothesised that ECM could direct this migration by providing directional cues or a scaffold for the neurons to migrate along. ECM components and receptors could also alter the morphology of the newborn neurons, as over-expression of integrin alpha 9 in human NPCs increased the outgrowth of neurites *in vitro* when grown on tenascin-C and when grafted into newborn rat cortex (Forbes and Andrews, 2019). Increased processes may be important for the interaction with the ECM environment, as discussed above for BPs (Kalebic et al., 2019).

ECM, once expressed, can be present in tissues for a long period of time (Brückner et al., 1998; Zimmermann and Dours-Zimmermann, 2008; Ewald, 2020). Therefore, the removal of ECM components at the right time is also important for neocortex development. Modulation of the ECM, such as degradation by metalloproteases (MMPs), has been reported throughout neocortex development. MMP2 has been shown to modulate the ECM deposited by growing microvessels within the early fetal human neocortex (Girolamo et al., 2004). This could impact neuronal migration, as BPs are reported to attach their basal process to blood vessel ECM later in neocortex development (Nowakowski et al., 2016), altering the scaffold of cell processes newborn neurons are able to migrate along. This builds a complex picture of ECM deposition and modulation that may help direct the migration of neurons and eventually determine the shape of the developing neocortex.

## ECM and Neurodevelopmental Disorders

It can be difficult to study the function of the ECM in human neurodevelopment, despite the recent advances in human cell- and tissue-based culture models. However, a lot has been learnt about the normal function of the ECM in neocortex development from various neurodevelopmental disorders. There are many mutations in ECM genes, or genes affecting ECM function, that lead to neurodevelopmental disorders, with RELN (for the protein Reelin) being one the most well-known examples.

**Figure 4:**
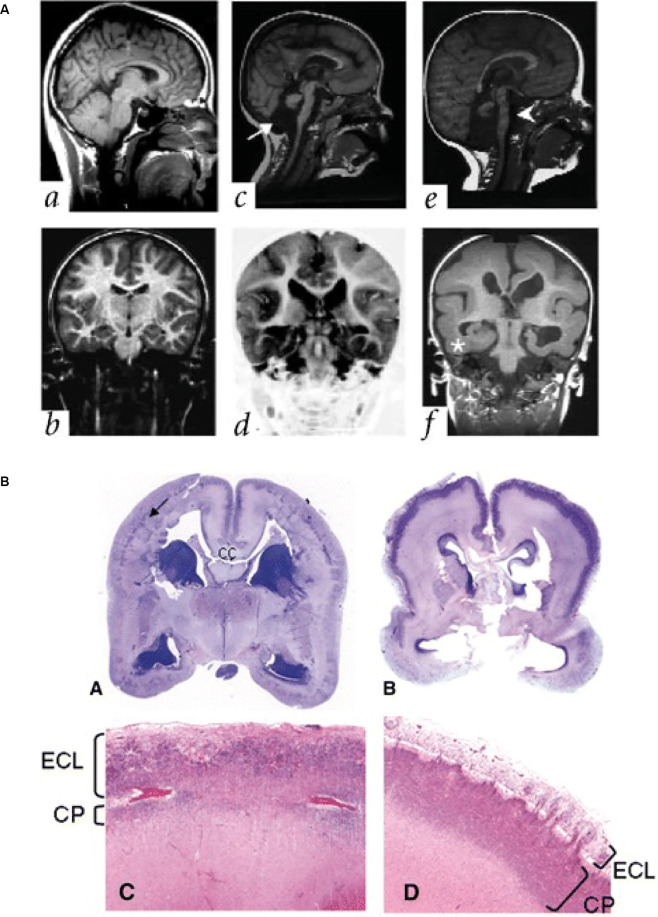
Lissencephaly in patients with mutations in ECM and ECM-associated genes. **(A)** Parasagittal (upper panels) and coronal (lower panels) MRI from patients with a normally developing brain (*a,b*), and two patients with lissencephaly associated with RELN mutations (*c,d*; *e,f*). Note the thickened cortex and reduced folding present. Adapted from Hong et al. (2000). **(B)** Images showing examples of cobblestone lissencephaly in a 19 pcw fetus with a POMT1 mutation (**A,C**, left panels) and 26 pcw fetus with a POMGNT1 mutation (**B,D**, right panels). The top panels show whole-mount sections of the brain. Arrow indicates impoverished cortical plate in the top left panel. The lower panels show higher magnification images of the cortical plate from these fetuses. Note the reduced size of the cortical plate (CP) and the thickened extracortical layer (ECL) in the lower left panel, but the thin ECL in the lower right panel. CC indicates the corpus callosum. Adapted from Devisme et al. (2012).

Reelin is an ECM glycoprotein (Hynes and Naba, 2012) secreted by several cell types, including the Cajal Retzius cells, early in the human fetal cortex (D’Arcangelo et al., 1995; Meyer et al., 2002). Mutations in the human gene RELN are linked to various neurodevelopmental disorders, including schizophrenia, bipolar disorder, and autism spectrum disorder (Ishii and Maeda, 2008; Lakatosova and Ostatnikova, 2012). RELN mutations are also known to cause type I lissencephaly, with abnormal lamination of the cortical plate and a loss of cortical folding (Figure 4A; Hong et al., 2000). The mechanisms underlying this malformation have been elucidated in mouse models (Hong et al., 2000; Fatemi, 2001; Rice and Curran, 2001; Pérez-Martínez et al., 2012; Sekine et al., 2012; Hirota and Nakajima, 2017; Mizukami et al., 2018), which show that Reelin is important for directing the migration of newborn neurons and the correct positioning of these neurons in the cortical plate.

The role of ECM in neuronal migration disorders is relatively common. Patients with another type of lissencephaly, cobblestone lissencephaly, also have mutations in genes that alter ECM function. In particular, the enzymes POMT1/2 that glycosylate the ECM receptor alpha-dystroglycan have been found in patients with cobblestone lissencephaly (Figure 4B; Devisme et al., 2012) and in Walker-Warburg syndrome (De Bernabé et al., 2002; Van Reeuwijk et al., 2005). Mutations in the genes that encode for the laminin subunits beta 2 and gamma 2 (LAMB2 and LAMC2) have also been associated with disorganisation of the cortical layers, similar to cobblestone lissencephaly (Radner et al., 2013), as have mutations in the laminin subunit beta 1 (LAMB1; Radmanesh et al., 2013). These mutations are thought to lead to these disorders *via* disruption of the pial basement membrane, which lies at the top of the cortical plate. This disruption enables migrating neurons to bypass their usual stop signals, migrating above the cortical plate and creating a bumpy, cobblestone-like appearance (Radner et al., 2013).

Mutations in laminin subunits are also linked to another folding disorder, polymicrogyria, characterised by an excess of shallower folds on the cortical surface. LAMC3 mutations have been found in patients with a developmental delay in the occipital cortex and focal areas of polymicrogyria (Barak et al., 2011). This laminin subunit was found to be highly expressed in the cortical plate at 20 post-conception weeks (pcw) human fetal cortex, with lower expression levels in the ventricular and inner and outer subventricular zones. This expression peaked around mid-gestation and was maintained until 12 months postnatally, coinciding with the peak of dendritogenesis and the generation of the majority of the cortical folds (Barak et al., 2011).

ECM has also been linked to other aspects of cortex shape during development and neurodevelopmental disorders, with several mutations in the collagen family found in patients. Mutations in collagen IV alpha 1 (COL4A1) have been linked to porencephaly, the development of a cavity in the cortex filled with cerebrospinal fluid (Hubert et al., 2009). COL18A1 mutations have also been found in patients with Knobloch syndrome and are associated with malformations of cortical development including heterotopias and pachygyria (Hubert et al., 2009). Both of these malformations are the result of abnormal neuronal migration, resulting in neurons clustering in abnormal layers in the cortical wall. Finally, COL1A1 and COL2A1 mutations have been associated with hydrocephaly, characterised by increased cerebrospinal fluid pressure and enlarged ventricles (Hubert et al., 2009). Together, this suggests an important role of ECM in shaping the developing human neocortex.

Many neurodevelopmental abnormalities are linked to much more focal malformations of cortical development, such as the abnormal lamination of neurons or dysmorphic neuronal cells, called balloon cells (Zamecnik et al., 2012). These malformations can be associated with epilepsy and are therefore often found in resected tissue from these patients. When tissue from such resections was examined, changes in the extracellular space were observed. These included an increase in the expression of the ECM components tenascin-C, tenascin-R, versican, and a decrease in brevican expression (Zamecnik et al., 2012).

Finally, there is also evidence that the modulation of the ECM in the developing neocortex is important for its development. Mutations in the enzyme neurotrypsin, a serine protease that degrades agrin, are linked to intellectual disability in a small number of patients (Molinari et al., 2002). Agrin is expressed during fetal neocortex development and is thought to help regulate synaptic function and be important for memory later on in life (Molinari et al., 2002). Several other ECM components are also known to have a vital role in perineuronal nets, which are also important for synaptic function (Barros et al., 2011; De Luca and Papa, 2016; Sorg et al., 2016; Miyata and Kitagawa, 2017). Perineuronal nets are lattice-like ECM structures that surround the synapses of certain neurons, including several interneurons (Sorg et al., 2016). They are predominately made up of chondroitin sulfate proteoglycans, tenascin, hyaluronan, and proteoglycan link proteins and hyaluronic acid (Sorg et al., 2016).

Impairment of perineuronal nets, in particular the enzyme that modifies them (matrix metalloproteinase-9), has been linked to several neurodevelopmental disorders, including fragile X syndrome, autism spectrum disorder, and epilepsy (Reinhard et al., 2015; Rogers et al., 2018; Wen et al., 2018). It has also been suggested that neuronal activity could feed back and modulate ECM expression surrounding neurons too (Lazarevich et al., 2020). As synaptic pruning occurs over the first few years of life in humans and non-human primates (Hensch, 2004), the ability to modulate the ECM that regulates synapse formation and stabilisation is vital to ensure this happens correctly (Gundelfinger et al., 2010).

There is growing evidence for the role of ECM in neurodevelopmental disorders, with more and more mutations in genes affecting ECM function discovered in patients. However, whilst we can associate the ECM with the cortical abnormalities detected, we still do not understand the mechanisms underlying them. Understanding the normal function of ECM in human neocortex development is an important step that would enable us to uncover how altering its functions leads to the malformations observed in these patients, potentially opening up new therapeutic avenues.

## What Else Can We Learn?

Many of the functions of the ECM in neocortex development have been uncovered using animal models (Barros et al., 2011; Faissner and Reinhard, 2015; Long and Huttner, 2019), but it remains unclear how many of these functions are conserved in humans. However, as we described above in the “ECM and Neural Progenitors” section, RNA sequencing studies have revealed key differences in ECM expression between these models and the developing human neocortex. As ECM components often interact with each other, functioning as a network, this change in ECM expression is likely to affect its function. This is one of the reasons why it is important we understand the role of the ECM in the development of the human neocortex.

Another reason is that many of the findings from development can be useful for understanding the role of the ECM in injury and neurodegeneration. For example, how ECM expression changes upon transplantation of iPSC-derived cells into the adult brain, how ECM composition is altered upon injury, and how this change may impact inflammation and repair (Roll and Faissner, 2014), which are all key issues for future research. ECM and ECM modulators are known to be expressed by reactive astrocytes but can also be expressed at lower levels by neurons, oligodendrocytes, microglia, and the cells of the vasculature. These include chondroitin sulphate proteoglycans, associated with the inhibition of axonal outgrowth, and heparan sulphate proteoglycans, associated with the promotion of axonal outgrowth. The balance of these two ECM families is vital for the repair of the adult neocortex (Roll and Faissner, 2014). Understanding their roles in human neocortex development could provide important information about this balance in the adult, informing and potentially improving current avenues for therapeutic options.

We therefore still have a lot to learn about the role of the ECM in human and non-human primate neocortex development. It is clear that ECM components are involved in almost every aspect of neocortex development, but exactly what ECM is expressed when and where is not yet known. Understanding these dynamics and the differences between species will be an important step forward in understanding how ECM shapes human neocortex development and the role it has played in the evolution of the human brain.

## Author Contributions

KL and WH wrote the manuscript. All authors contributed to the article and approved the submitted version.

## Conflict of Interest

The authors declare that the research was conducted in the absence of any commercial or financial relationships that could be construed as a potential conflict of interest.

## Publisher’s Note

All claims expressed in this article are solely those of the authors and do not necessarily represent those of their affiliated organizations, or those of the publisher, the editors and the reviewers. Any product that may be evaluated in this article, or claim that may be made by its manufacturer, is not guaranteed or endorsed by the publisher.
